# The importance of trust in the relation between COVID-19 information from social media and well-being among adolescents and young adults

**DOI:** 10.1371/journal.pone.0282076

**Published:** 2023-03-23

**Authors:** Adam J. Hoffman, Luke McGuire, Channing J. Mathews, Angelina Joy, Fidelia Law, Marc Drews, Adam Rutland, Adam Hartstone-Rose, Mark Winterbottom, Kelly Lynn Mulvey

**Affiliations:** 1 Department of Psychology, Cornell University, Ithaca, New York, United States of America; 2 Department of Psychology, University of Exeter, Exeter, United Kingdom; 3 Department of Psychology, University of Virginia, Charlottesville, Virginia, United States of America; 4 Department of Psychology, North Carolina State University, Raleigh, North Carolina, United States of America; 5 EdVenture, Columbia, South Carolina, United States of America; 6 Department of Biological Sciences, North Carolina State University, Raleigh, North Carolina, United States of America; 7 Faculty of Education, University of Cambridge, Cambridge, United Kingdom; IIM Bodh Gaya: Indian Institute of Management Bodh Gaya, INDIA

## Abstract

During the COVID-19 pandemic, young people have been exposed to distressing content about COVID-19 without knowing whether they can trust such content. This indicates a need to examine the effects of social media use on mental health and well-being. Existing research provides an inconsistent impression of such effects. Thus, we examined the relation between exposure to COVID-19 information on social media and well-being and assessed if trust in COVID-19 information on social media moderated this relationship. The sample consisted of 168 adolescents and young adults from the U.K. and U.S. (*M*_age_ = 17.4 years). Participants completed measures of exposure to, and trust in, COVID-19 information on social media platforms, and measures of emotional, psychological, and social well-being. Results revealed a null to positive relation between exposure to COVID-19 information on social media and well-being across measures. However, when trust was added to the models as a moderator, results indicated that, for adolescents with higher levels of trust in COVID-19 information found on social media, the relation between information encountered on social media and well-being was positive. In contrast, for adolescents with lower levels of trust, the association between information encountered on social media and well-being was null or sometimes negative. Given the lack of consensus about the impact of social media use on well-being, these results point to the importance of trust when assessing the relationship between exposure to COVID-19 information and well-being.

## Introduction

During the COVID-19 pandemic, social media outlets were flooded with information and misinformation regarding the COVID-19 virus and the long-reaching negative effects of the virus on our everyday lives, social interactions, and general well-being [1, 2]. These continuous stories became inescapable to almost anyone consuming media. Indeed, terms like “doomscrolling” (continuous viewing of information that was emotionally and/or psychologically taxing) or “news avoidance” (purposefully avoiding stressful information) became colloquial and commonly discussed phenomena when considering the impact of continuous streaming of COVID-19 information on people’s mental health and well-being [3].

Unlike most other historical pandemics in the West, the COVID-19 virus spread across nations in a highly globalized world. This globalization allowed for information (both factual and counterfactual) to be easily accessed and spread through and across many types of media, with social media heightening concern internationally [4]. Some information about COVID-19 shared through social media was false and termed as “misinformation” [5]. The introduction of misinformation only exacerbated existing distrust of information viewed on social media platforms, especially among younger age demographics [6].

Adolescents and young adults consume social media more than any other media outlet [7, 8]. The effects of such use have been investigated and discussed extensively in popular media across prominent online and traditional media news (e.g., [9]). However, research to date has not fully examined the effects of social media use on adolescents and young adults during the global COVID-19 pandemic. Furthermore, given the decaying trust of information on social media [6], more attention is needed to understand how adolescent and young adult trust in social media could impact the role of social media in their lives. Thus, we investigated the relation between encountering COVID-19 information on social media platforms and the emotional, psychological, and social well-being of adolescents and young adults, whilst considering the role of trust as a potential moderator.

### Social media use and implications for well-being

In 2021, a record high number of 72% of adults engaged with at least one social media site, with younger adults being more likely than older adults to have a social media account across multiple platforms [8]. There are similar statistics in the UK, as 78% of adults engage in some form of social media use [10]. Not only are a vast majority of adults on social media, many are frequent users. For example, 49% of Facebook users and 30% of Twitter users report visiting these apps or sites multiple times each day [8]. In the UK, adults are spending an average of 16.6 hours on Facebook and 19.9 hours on TikTok each month [10]. Among American adolescents 97% report using at least one social media site and 95% report ownership or access to a smartphone or computer to view social media [11]. In the UK, 87% of adolescents report using at least one social media site and 91% report that they own or have access to a smartphone or computer to view social media [12].

Given the increase in social media usage, researchers have investigated its impact on the mental health and well-being of young people, and have found both positive *and* negative affect influences on well-being [13]. Among qualitative studies, research reveals that young people perceive social media as positive as it can foster social connections, make it easier to find news and information, and provide inspiration for exploration of their identities and affirm self-expression [11, 13]. Conversely, young people also perceive social media use to be negative detailing that it facilitates bullying and rumor spreading, envy or inferiority, spread of misinformation, and boredom/wasted time, all of which could lead to poorer mental health outcomes [13, 14].

Indeed, quantitative research corroborates these mixed effects of social media use. For example, one longitudinal study of adolescents aging into early adulthood revealed no significant association between social media use and anxiety or depression over the eight years of the study [15]. However, among college-attending emerging adults, other researchers found a positive effect of social media use upon psychological well-being due to the positive effect of bonding and social capital [16]. Conversely, in a 5-year longitudinal study, greater use of social media earlier in adolescence was associated with greater decreases in well-being, compared to adolescents who engaged in less social media use [17]. Specific to the era of COVID-19, one study with very young American adults who have a history of adverse childhood experiences found a positive association between getting COVID-19 information via social media and elevated depression and PTSD symptoms [18]. In another study with a predominately Emitrai adolescent and adult sample revealed that increased social media use during the COVID-19 pandemic was positively associated with poorer well-being [19]. Some studies suggest that social media use may be particularly detrimental for adolescent girls (e.g., [17]), while others have observed no effect of gender (e.g., [15]).

Well-being has become an increasingly significant outcome for clinical psychology research as there has been a renewed push through positive psychology to understand mental health beyond psychological dysfunction, and to also understand what factors facilitate and even promotes psychological wellness [20]. It is important to note that well-being is theorized to be comprised of three dimensions or parts; emotional, psychological, and social well-being [21]. Emotional well-being is comprised of the judgment of life satisfaction and general positive affect. Psychological well-being captures positive functioning that assesses self-acceptance, personal growth and autonomy. Finally, social well-being captures well-being as it relates to social integration, acceptance, actualization, and coherence. The relation between social media use and social and emotional well-being may be particularly strong, compared the psychological well-being, as social media use is likely to have more direct impacts on social and emotional well-being given the highly social and affective nature of social media. Further, given the documented differential impact of social media on the social and emotional lives of individuals [22, 23], it is important to consider these dimensions independently as their relations may differ.

### Trust in social media

One critical factor that could explain inconsistencies in the findings above, and provide a better understanding of the role of social media use in the well-being of adolescents and young adults, is trust [24]. This factor could be particularly important when considering *information* or *news* that adolescents are consuming on social media. There is good reason to believe that young people may not trust information they are viewing on social media. The Edelman Trust Barometer Reports revealed that for years and across the globe, individuals’ trust in many institutions across society has been waning (e.g., government, businesses, non-governmental organizations), with media representing the least trusted social institution [6]. Trust in media was further eroded during the COVID-19 pandemic with the spreading of misinformation on social media, in what the World Health Organization termed an “Infodemic” (a time of unprecedented and excessive amounts of misinformation that make it difficult to identify reliable sources of information) [25, 26]. In 2021, trust in information from the media was rated a record low, with social media representing the least trusted form [6]. Among adults across the globe, 44% report trusting news (overall), whereas only 26% believe the news they see on their social media [26].

Given the precipitous drops in trust, understanding trust may be a particularly important factor in understanding the complex relationship between encountering information on social media and well-being. Indeed, trust in social media has been shown to be an important moderator and mediator of the effects of social media on other outcomes. For example, research has revealed that the extent to which an individual trusts a social media platform can impact the relationship between social media exposure and (mis)information building (e.g., believing in conspiracy theories) [27]. Specifically, for individuals who have greater trust in social media, there was a stronger positive association between social media exposure and belief in conspiracy theories and misinformation, compared to those who had less trust [27]. In a study examining more global, institutional trust (including media) during the early COVID-19 pandemic among adults, results indicated that greater trust was associated with more happiness, greater life satisfaction, and better mental health [28]. Other research has also found a positive association between trust in general media and positive well-being among adults during the COVID-19 pandemic [29]. In our search, we found one study that examined adolescents’ trust in social media and the results indicated no association between trust in social media and health literacy about COVID-19 [30].

The limited research around trust in social media among adolescents and young adults represents a significant gap in the literature given the extent of social media use in this age range. The predictions of the current study are derived from the Uncertainty Management Framework (UMF) [31], which argues that individuals seek information to suppress uncertainties and stress caused by their health concerns. When information is trustworthy, it is a useful tool that can help us to cope with the stress derived from health concerns, such as concerns over COVID-19. However, when this trust is diminished, this information is less useful as a coping strategy. Brashers notes that coherence is an important component of using information to cope, with incoherent information (such as may be found on social media and exacerbated by the presence of misinformation) leading to uncertainty and stress. One recent study supports this theory, as research revealed trust in media to be a significant mediator in the relation between news exposure and perceived gratitude and satisfaction during the COVID-19 pandemic, such that those who had greater trust reported greater gratitude and satisfaction when exposed to news media [29]. While this work is an important first step, this study used an adult, primarily White, sample from the US collected via MTurk. Thus, additional research is needed that explores these relations with key populations where social media may be influential—namely adolescents and young adults from multiple countries with greater attention to sampling a population with ethnic and racial diversity.

Some social media platforms are perceived as spreading misinformation about COVID-19. Indeed, among adults in the UK and US, Facebook and Twitter are viewed as the most concerning platforms for the spreading of misinformation [26]. Though the short video social media platform, TikTok, does not facilitate the sharing of news information in similar ways to Facebook or Twitter, it became very popular among adolescents and young adults during the COVID-19 pandemic with a 180% increase in users from 15–25 years old after the onset of the COVID-19 pandemic [32]. TikTok developed a reputation for facilitating the spread of misinformation among its users, particularly among its adolescent users [33]. Thus, we believe that it is important to consider each of these social media platforms, and their relationship to well-being, independently given the differences in news information delivery and format of these social media platforms and the reported differing levels of trust in each of them. Indeed, prior research has also demonstrated the importance of considering effects of social media platforms individually as their relations to well-being can be significantly different based on the use of different platforms [34].

### The current study

Given the aforementioned gaps, we investigated three questions. First, what was the association between the frequency of exposure to COVID-19 information on social media and well-being among adolescents and young adults? Given the uncertainty and often troubling news about the COVID-19 pandemic that was frequently shared on social media, we expected that there would be a negative association between the frequency of encountering COVID-19 information and well-being (Hypothesis 1). Second, what is the association between trust in COVID-19 information and well-being? We expected a positive association between trust in COVID-19 information and well-being (Hypothesis 2). Our third question asks, does trust moderate this relation between frequency of encountering COVID-19 information and well-being among adolescents and young adults? For adolescents and young adults with high and average levels of trust, we expected the association between the frequency of encountering COVID-19 information and well-being would be null or positive. Though some information about the COVID-19 pandemic was likely challenging to a sense of well-being, adolescents and young adults may have been able to find solace and comfort as they trusted the information they were consuming [31]. Alternatively, for adolescents and young adults with low levels of trust, we expected a negative association between the frequency of encountering of COVID-19 information and well-being, as adolescents and young adults may experience significant stress if they are consuming information but do not trust the source of the information (Hypothesis 3) [29].

## Methods

### Participants

The sample consisted of 168 adolescents and young adults (125 girls and 43 boys; *M*_age_ = 17.4 years, SD = 1.79) from the United Kingdom (*n* = 93) and the United States (*n* = 75). A majority of the participants were between the age of 14 to 18 years old (*n* = 128; 76.2%) and the remaining participants ranged in age from 19 to 23 years old (*n* = 40; 23.8%). Participants reflected the ethnic-racial diversity of their communities, with 38.0% White British or European American, 25.6% South Asian British or Asian American, 10.2% Black British or African American, 1.7% Hispanic or Latino, 9.6% Bi-racial, and 12.5% self-identified as other. Lastly, 2.4% of participants chose not to report their ethnicity. Participants were recruited through informal STEM learning sites (ISLS), where they participated in youth educator programs that involved learning about STEM content and communicating this content to the visitors (for instance, by staffing interactive activities). Participants were drawn from the UK and US as these were two countries that significantly impacted by both COVID-19 pandemic but also misinformation about COVID-19 on social media platforms. In 2020, both the UK and the US were ranked among the top 10 countries most affected by the spread of the COVID-19 [35]. Misinformation plagued social media in both countries but it was particularly prevalent in the US compared to other countries [36].

### Procedure

The study was approved by the Institutional Review Boards at North Carolina State University and the University of Exeter. Families were notified of the study and only potential participants with written informed parental consent (or who consented themselves if they were over 18) participated in the study. Further, all adolescents assented and all young adults consented to participation in a written format before completing the survey. Participants were compensated with a small electronic gift card for the completion of the study survey. All participants completed the survey in May or June of 2020, toward the beginning of the COVID-19 pandemic in the U.K. and U.S. Participants completed the surveys independently on a computer via Qualtrics.

### Measures

#### Frequency of exposure to COVID-19 information on social media platforms

One item was used to assess the frequency of COVID-19 information that participants were exposed to on a given social media platform. Participants responded to this item for an array of social media platforms, including Facebook, TikTok, and Twitter: How often do you encounter information about COVID-19 from <social media platform>?). Responses were rated on 7-point scales (1 = *Never*; 7 = *Everyday*) for each of the three of the social media platforms assessed in the study. Given the aforementioned potential differences in social media use around various platforms, including Facebook, TikTok, and Twitter, it was important to assess the frequency of exposure to COVID-19 information by each specific social media platform.

#### Trust in COVID-19 information on social media

One item assessed participants’ trust in information about COVID-19 that they encountered across various social media platforms (e.g., How much do you trust the information you encounter on social media about COVID-19?). The item was rated on a 7-point scale (1 = *Don’t trust at all*; 7 = *Trust completely*).

#### Well-being

Participants completed The Mental Health Continuum–Short Form [37] to assess well-being. The measure is comprised of three subscales that measure emotional well-being, social well-being, and psychological well-being. Emotional well-being was assessed with three items (e.g., During the past month how often did you feel interested in life?); social well-being with five items (e.g., During the past month how often did you feel that you belonged to a community?); and psychological well-being with six items (e.g., During the past month how often did you feel confident to think or express your own ideas?). Adolescents rated agreement on a 6-point scale (1 = *Never*; 6 = *Everyday*), and items were averaged to create a mean score for each of the subscales, with higher scores indicating greater well-being. Measures of emotional, social, and psychological well-being had strong reliability, Cronbach α values were .88, .78, and .88, respectively. This measure has been assessed and validated with adolescent and adult samples from many different countries including the U.K. and the U.S. (see [38, 39] for reviews). See S1 Appendix for a full list of items for all measures used in the study.

### Analysis plan

All analyses were conducted in SPSS 27. First, means and bivariate correlations were computed and assessed to determine patterns and associations among key study variables. Then moderation analyses were conducted with the PROCESS Macro Version 3.5 [40]. To assess moderation, a series of multiple regression models were calculated to assess whether trust in COVID-19 information moderated the relation between the frequency of COVID-19 and well-being across three different social media platforms. In the models, the two covariates, Frequency of Encountering COVID-19 information and Trust in COVID-19 information were mean-centered and entered into the models. In addition, an interaction term of these two covariates was calculated and added to the model. If a significant interaction effect was detected, the interaction was probed to more easily interpret the effect of the interaction. Given the relatively wide age range of the sample and the proportional imbalance of gender in our sample, age and gender were included in all models as control variables. Though no differences in the associations were expected to be different on the basis of country of origin (U.S. or U.K.), to be more conservative, we elected to also include country of origin as a control variable in all models.

## Results

### Descriptive statistics

To develop a better understanding of the data, descriptive statistics were computed (See Table 1). Means for COVID-19 information frequency revealed that participants encountered COVID-19 information on social media platforms at least a few times a week, rather than every day. A one-way ANOVA revealed significant differences in the frequency of COVID-19 information encountered across the social media applications (*F*(2, 316) = 4.34, *p* = .01, η_p_^2^ = 0.03). Pairwise posthoc comparisons with Bonferroni corrections suggested that participants encountered significantly more COVID-19 information on Twitter than on Facebook (3.48 vs. 2.80, *p* = .01).

**Table 1:** Means, sample sizes, and zero-order correlations for key study variables.

	*M* (SD)	*n*	1	2	3	4	5	6	7
1. COVID-19 Information Frequency–Facebook	2.87 (2.37)	164	-						
2. COVID-19 Information Frequency–TikTok	3.16 (2.34)	162	.21**	-					
3. COVID-19 Information Frequency–Twitter	3.47 (2.46)	161	.31**	.22**	-				
4. Trust in COVID-19 Information in Social Media	2.93 (1.40)	164	.02	.18*	.25**	-			
5. Emotional Well-being	3.84 (1.20)	162	.05	.11	-.10	.04	-		
6. Psychological Well-being	3.30 (1.03)	164	.13	.12	-.04	.04	.60**	-	
7. Social Well-being	4.20 (1.08)	164	.09	.17*	-.08	.23**	.63**	.64**	-

Note: *p < .05

**p < .01.

Among bivariate correlations of the key variables, results indicated that greater frequency of encountering COVID-19 information on one platform of social media was related to greater frequency of encountering COVID-19 information on other social media platforms (*r*’s < .21, *p*’s < .01). Greater frequency of encountering COVID-19 information on TikTok and Twitter was related to greater trust in COVID-19 information from the respective social media platform (*r*’s < .18, *p*’s < .01), but not for Facebook (*r* = .02, *p* = .80). Finally, greater trust in social media information about COVID-19 was related to better social well-being (*r* = .23, *p* < .01), but not emotional or psychological well-being (*r* = .04, *p* = .66; *r* = .04, *p* = .64, respectively).

### Moderation analyses

In total nine moderation models were estimated to assess the moderating effect of trust of COVID-19 information from social media on the relation between three social media platforms (Facebook, TikTok, and Twitter) and across three different well-being outcomes (emotional well-being, psychological well-being, and social well-being). See Tables 2–4 for all model estimates. Results indicated that frequency of encountering COVID-19 information on TikTok was positively associated with all three well-being outcomes. Frequency of encountering COVID-19 information on Facebook or Twitter was not associated with any of the well-being outcomes. The main effects of trust in COVID-19 information with social well-being outcomes were significant across the three social media platforms. No significant main effects were observed for trust in COVID-19 information and emotional or psychological well-being.

**Table 2:** Estimates for the main effects and interaction effects of frequency of COVID-19 information on Facebook and trust in COVID-19 information on well-being.

Variable	*B*	*(S*.*E*.*)*	*95% Confidence Interval for B*		β
			Lower bound	Upper bound	
Emotional Well-being					
Frequency of COVID-19 information on Facebook	.05	(.04)	-.02	.12	.07
Trust in COVID-19 information	-.03	(.06)	-.16	.09	-.02
Frequency of COVID-19 information on Facebook × Trust in COVID-19 information	.09**	(.03)	.04	.14	.26
Psychological Well-being					
Frequency of COVID-19 information on Facebook	.07*	(.03)	.00	.14	.14
Trust in COVID-19 information	-.02	(.06)	-.14	.09	-.02
Frequency of COVID-19 information on Facebook × Trust in COVID-19 information	.11**	(.02)	.06	.15	.35
Social Well-being					
Frequency of COVID-19 information on Facebook	.06*	(.03)	.00	.12	.10
Trust in COVID-19 information	.12*	(.05)	.01	.22	.19
Frequency of COVID-19 information on Facebook × Trust in COVID-19 information	.09**	(.02)	.05	.13	.29

*Note*. Controls included adolescent gender, age, and country of residence. * *p* < .05. ** *p* < .01

**Table 3:** Estimates for the main effects and interaction effects of frequency of COVID-19 information on TikTok and trust in COVID-19 information on well-being.

Variable	*B*	*(S*.*E*.*)*	*95% Confidence Interval for B*		β
			Lower bound	Upper bound	
Emotional Well-being					
Frequency of COVID-19 information on TikTok	.08*	(.04)	.00	.16	.10
Trust in COVID-19 information	-.02	(.07)	-.15	.11	.01
Frequency of COVID-19 information on TikTok × Trust in COVID-19 information	.07**	(.03)	.02	.12	.23
Psychological Well-being					
Frequency of COVID-19 information on TikTok	.07*	(.04)	.00	.15	.11
Trust in COVID-19 information	-.01	(.06)	-.13	.11	.00
Frequency of COVID-19 information on TikTok × Trust in COVID-19 information	.07**	(.02)	.02	.11	.24
Social Well-being					
Frequency of COVID-19 information on TikTok	.09*	(.03)	.02	.15	.13
Trust in COVID-19 information	.13*	(.06)	.02	.24	.21
Frequency of COVID-19 information on TikTok × Trust in COVID-19 information	.05*	(.02)	.01	.09	.19

*Note*. Controls included adolescent gender, age, and country of residence. * *p* < .05. ** *p* < .01

**Table 4:** Estimates for the main effects and interaction effects of frequency of COVID-19 information on Twitter and trust in COVID-19 information on well-being.

Variable	*B*	*(S*.*E*.*)*	*95% Confidence Interval for B*		β
			Lower bound	Upper bound	
Emotional Well-being					
Frequency of COVID-19 information on Twitter	-.04	(.04)	-.11	.04	-.14
Trust in COVID-19 information	.04	(.07)	-.09	.18	.09
Frequency of COVID-19 information on Twitter × Trust in COVID-19 information	.07*	(.03)	.01	.13	.22
Psychological Well-being					
Frequency of COVID-19 information on Twitter	-.01	(.04)	-.08	.06	.04
Trust in COVID-19 information	.02	(.06)	-.10	.15	.06
Frequency of COVID-19 information on Twitter × Trust in COVID-19 information	.08**	(.03)	.03	.13	.03
Social Well-being					
Frequency of COVID-19 information on Twitter	-.05	(.03)	-.12	.01	-.17
Trust in COVID-19 information	.19**	(.06)	.08	.31	.29
Frequency of COVID-19 information on Twitter × Trust in COVID-19 information	.07**	(.02)	.03	.12	.24

*Note*. Controls included adolescent gender, age, and country of residence. * p < .05. ** p < .01

**Fig 1:**
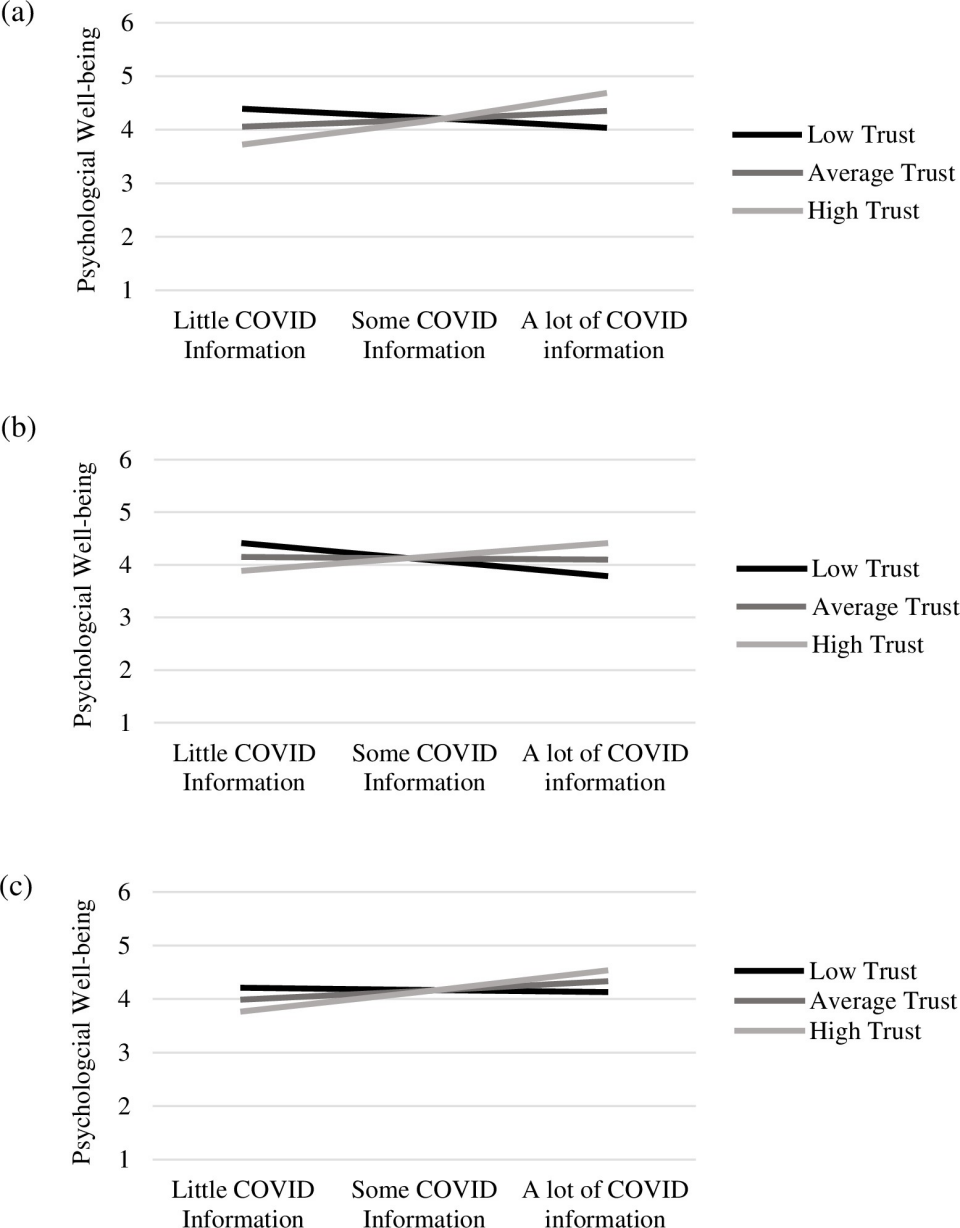
Moderation Effect of Trust of COVID-19 Information on the Relation between Frequency of COVID-19 Information on (a) Facebook, (b) TikTok, and (c) Twitter with Psychological Well-being. *Note*. Solid lines indicate a significant effect and dashed lines indicate a non-significant effect.

Across the nine moderation models estimated, a significant positive interaction effect was observed, with similar general trends across the models. For illustrative purposes, we have plotted the probed interactions across the three social media platforms and psychological well-being (see Fig 1). For the figures with emotional and social well-being, see S2 Appendix. Conditioned values of trust in COVID-19 information were set to the mean and +/- one standard deviation of the mean. Across all interactions, with the exception of COVID-19 information from Twitter and emotional and social well-being, frequency of encountering COVID-19 information was positively associated with well-being for participants with greater trust in COVID-19 information (*b*s ≥ 0.10, *p*s ≤ .04). A positive effect for frequency of encountering COVID-19 information with well-being was also observed for adolescents and young adults with average levels of trust across all well-being measures for TikTok (*b*s ≥ 0.07, *p*s ≤ .045) and for psychological and social well-being for Facebook (*b* = 0.07, *p* = .04; *b =* 0.06, *p =* .04, respectively). For adolescents and young adults with low trust in COVID-19 information, the frequency of encountering COVID-19 information on Twitter was negatively associated with all well-being outcomes (*b*s ≤ -0.13, *p*s ≤ .02) and was trending toward a significant association for frequency of encountering COVID-19 information on Facebook and psychological well-being (*b* = -0.08, *p* = .08). No significant effects were observed for adolescents and young adults with low trust between the frequency of encountering COVID-19 information on TikTok and the well-being outcomes (*b*s ≤ -0.02, *p*s ≥ .71).

## Discussion

Results across the three social media platforms suggested that there was a null or small positive relation between the frequency of COVID-19 information encountered on social media and well-being. However, a more nuanced story unfolded with the inclusion of trust in COVID-19 information as a moderator of this relation. Results indicated that trust in information is a driving factor in the directionality of this relation, such that for adolescents and young adults who trusted information, the positive relation between information encountered and well-being was stronger. Conversely, for adolescents and young adults who did not trust information, the positive relation between information encountered and well-being was null, or in some cases, a negative relation. We discuss the implications of these results and conclude with limitations of the study and promising possibilities for future research on these topics.

### Encountering information on social media and well-being

Contrary to our expectations (Hypothesis 1), we observed positive and null associations between frequency of encountering COVID-19 information and well-being. Given the conflicting and typically troubling news and information around COVID-19 at the time when data were collected (April and May 2020, when cases were becoming increasing widespread for the first time), we reasoned that the more COVID-19 information adolescents encountered the poorer well-being they would report. In the case of Twitter, frequency of encountering COVID-19 information was not associated with well-being and, for Facebook and TikTok, it was positively associated with well-being. It is important to note that the positive associations that were observed had small effect sizes, suggesting that the positive associations observed were small in magnitude. One possible explanation for this positive relation between frequency of encountering COVID-19 information and well-being could be that exposure to more COVID-19 information, albeit likely difficult and depressing, offers a sense of feeling more informed and having a better understanding of the events of the world. Thus, encountering information and feeling better informed could be related to greater positive well-being, compared to those who do not encounter information about relevant and imminent events in the world, like the COVID-19 pandemic. This notion of more information leading to greater well-being is theorized to be the case for individuals who are already experiencing serious illness [41]. We argue that this logic could be extended to individuals who aim to avoid serious illness, like COVID-19. Indeed, individuals do not need to have already contracted COVID-19 to perceive greater well-being because they have more information about COVID-19, whether it be how to avoid it or treat it if contracted.

Our results contribute to the relatively muddled evidence regarding this relation between social media use and well-being and mental health. With mounting evidence indicating an array of positive, negative, and null relations of social media exposure with well-being, it is becoming increasingly clear that assessing the direct relationship between social media exposure/use and well-being may be of limited use and will provide inconsistent results [42, 43]. We argue, along with other scholars, for a more nuanced understanding on this relationship, which accounts for moderators and mediators, and which therefore provides better understanding of the conditions in which relations do and do not exist and in what direction [22].

### Trust and well-being

Results provided partial support for Hypothesis 2, as greater trust in COVID-19 information seen on social media was associated with social well-being, but was unrelated to emotional or psychological well-being. We expected that enhanced trust in information found in social media would be an indicator of general psychological adjustment and subsequently would be related to greater well-being. In retrospect, perhaps the null association between trust in social media COVID-19 information with emotional and psychological well-being is not unexpected given the recent accounts of mistrust around information gleaned from social media. The positive association between trust in COVID-19 information and social well-being was relatively robust and consistent across the social media platforms. Conceivably, the reason this association was stronger for social well-being, compared to the other dimensions of well-being, is because adolescents and young adults who view social media as trustworthy are probably more likely to engage and be exposed to social media, thus offering a stronger sense of social connectedness and well-being. Indeed, this reasoning is supported by other research examining related trust and well-being measures. For example, researchers found that greater trust in the government to deal with financial and health difficulties during the COVID-19 pandemic was positively associated with satisfaction with life and well-being [44]. Our results contribute to a burgeoning body of research suggesting that greater trust in the institutions of society is related to greater and more positive well-being among individuals, specifically with social well-being [44–46].

### The importance of trust on the implications of social media

To provide a more nuanced understanding of the relation between exposure to COVID-19 information on social media and well-being, we examined the role of trust in COVID-19 information encountered on social media, as a moderator. General trends across the social media platforms supported our hypothesis (Hypothesis 3). The extent to which adolescents trusted COVID-19 information is important as it predicted the directionality of the relation between exposure to COVID-19 information on social media and well-being. Specifically, for adolescents with greater trust, a positive association between exposure to COVID-19 information on social media and well-being was observed. In contrast, for adolescents and young adults with lower trust, the relation between exposure to COVID-19 information on social media and well-being was null or even negative. The negative relation was particularly pronounced when considering the specific social media platforms of Twitter and Facebook (though this negative relation was only trending toward significance for Facebook). Both of these platforms and the companies behind them have developed reputations for spreading misinformation [7, 8]. Perhaps this perception of these social media platforms as not trustworthy and facilitators of spreading misinformation is the reason why the relationship between exposure to COVID-19 information and well-being is negative for adolescents with lower levels of trust. Although lack of trust in COVID-19 information on social media could have negative implications for well-being, research has begun to examine how trust also plays a role in the spreading of misinformation on social media. One study found that greater trust in social media contributed to increasing belief in COVID-19 myths and conspiracies, which then lead to less critical social media posting practices and more spreading of misinformation [47]. Thus, some skepticism in information found on social media can provide benefits on a societal level, however this skepticism could potentially come at the cost of the individuals’ well-being.

Finally, our results lend credence to the larger notion that trust is an important concept when considering the effects and influence of most kinds of information encountered on social media for adolescents and young adults [48]. Indeed, research on other forms of information found on social media outside of COVID-19 information (e.g., tourism/travel information or diet information) has also revealed that trust can be a factor in the interpretation and influence of information encountered on social media platforms [49, 50].

### Limitations and future directions

Though this study is characterized by several strengths, it is important to consider the limitations of the study. First, the data that were used for the study were cross-sectional and thus, causation cannot be determined. However, the study provides foundational evidence, and highlights the need for future research to examine further exposure to information on social media and well-being with longitudinal data and explore the importance of trust of information in that relationship. Moreover, some single-item measures were used. We would encourage future research to develop scales related to the use of and trust in social media to enhance our understanding of these factors. Our sample could also be considered a limitation of the study. Adolescents were recruited from extracurricular programing aimed at enhancing interest in science, technology, engineering, and mathematics, thus these youth could have greater awareness of science literacy and misinformation around the science of COVID-19, thereby exacerbating the negative relation between exposure to COVID-19 information and well-being. Also, although the ethnic-racial diversity of the sample did reflect that of the two countries that adolescents were recruited from, there was an imbalance of gender in the study, where girls were overrepresented. Though we did control for the effect of gender in our study, future studies should recruit more representative samples to ensure the generalizability of these findings.

Future research on this topic is encouraged to consider other factors that may also moderate this relation, in addition to assessing trust as a moderator. Inclusion and assessment of the multiple moderators will ensure the robustness of the effects and increase the generalizability of these findings. An important consideration for future research could be to better understand these relations within individuals who actively seek out information and want to learn more versus individuals who actively avoid information. Indeed, one might expect negative relation between information encountered on social media and well-being individuals who work to actively avoid information but a positive relation for those who actively search for information. Finally, future research in this area should consider the extent which individuals perceive misinformation to be common to a social media platform. Though an individual may have trust in information shared on social media broadly, the reputation for or perception of misinformation for specific platforms could also impact the relation between information encountered on social media and well-being. In addition to the assessment of moderating factors of the relation between exposure to COVID-19 information on social media and well-being, we encourage future researchers to also consider mediating factors as well, to help better understand what could be explaining this relation. For example, interest in understanding COVID-19 would likely be an important mediator impacting this relation.

## Conclusions

Since the inception of social media in our society, we have wondered about its effect on our well-being, particularly among adolescents, some of the most frequent users of social media. Yet, despite over a decade of research, there is little consensus as to whether or not social media is actually detrimental or enhances our well-being. Given this lack of clarity, we aimed to provide a clearer understanding of this relation by examining it through a lens of trust as it relates to encountering COVID-19 information early in the COVID-19 pandemic. Our results provided evidence that trust is a critical factor when considering the relation between exposure to social media and well-being among adolescents and young adults. Though our results do suggest that having enhanced trust in the information adolescents are consuming can be beneficial for their well-being, we caution and emphasize the importance of strong skills, like critical thinking, analysis, and problem solving, in deciphering creditable sources of information. We encourage schools and universities to actively enhance these skills with adolescents and young adults, especially on social media where they are likely to encounter and engage with information, so that the information that is gleaned is accurate but also perceived as trustworthy.

## Supporting information

S1 Appendix Individual items for primary study measures. (DOCX)

S2 Appendix Moderation Effect of Trust of COVID-19 Information on the Relation between Frequency of COVID-19 Information on (a) Facebook, (b) TikTok, and (c) Twitter with Emotional Well-being. (DOCX)
