# Supplementary material for: The importance of trust in the relation between COVID-19 information from social media and well-being among adolescents and young adults
Source: PLoS One. 2023 Mar 23;18(3):e0282076. doi: 10.1371/journal.pone.0282076 (PMC10035839; doi:10.1371/journal.pone.0282076)
Supplement: S1 Appendix — (DOCX) [file pone.0282076.s001.docx]

**Supporting Information**

**Appendix A**

**Individual Items for Primary Study Measures**

*Frequency of Exposure to COVID-19 Information on Social Media Platforms*

1. How often do you encounter information about COVID-19 from Facebook?
2. How often do you encounter information about COVID-19 from TikTok?
3. How often do you encounter information about COVID-19 from Twitter?

*Trust in COVID-19 Information on Social Media Platforms*

1. How much do you trust the information you encounter on social media about COVID-19?

*Well-being*

Emotional well-being

1. During the past month, how often did you feel happy?
2. During the past month, how often did you feel interested in life?
3. During the past month, how often did you feel satisfied with life?

Social well-being

1. During the past month, how often did you feel that you had something to contribute to society?
2. During the past month, how often did you feel that you belonged to a community (like a social group, school, neighbourhood, etc.)?
3. During the past month, how often did you feel that our society is a good place, or is becoming a better place, for all people?
4. During the past month, how often did you feel that people are basically good?
5. During the past month, how often did you feel that the way our society works made sense to you?

Psychological well-being

1. During the past month, how often did you feel that you liked most parts of your personality?
2. During the past month, how often did you feel good at managing the responsibilities of your daily life?
3. During the past month, how often did you feel that you had warm and trusting relationships with others?
4. During the past month, how often did you feel that you had experiences that challenged you to grow and become a better person?
5. During the past month, how often did you feel confident to think or express your own ideas and opinions?
6. During the past month, how often did you feel that your life has a sense or direction or meaning to it?
