# Supplementary material for: The importance of trust in the relation between COVID-19 information from social media and well-being among adolescents and young adults
Source: PLoS One. 2023 Mar 23;18(3):e0282076. doi: 10.1371/journal.pone.0282076 (PMC10035839; doi:10.1371/journal.pone.0282076)
Supplement: S2 Appendix — (DOCX) [file pone.0282076.s002.docx]

**Supporting Information**

**Appendix B**

**Moderation Effect of Trust of COVID-19 Information on the Relation between Frequency of COVID-19 Information on (a) Facebook, (b) TikTok, and (3) Twitter with Emotional Well-being**

(a)

(b)

(c)

*Note.* Solid lines indicate a significant effect and dashed lines indicate a non-significant effect.

**Moderation Effect of Trust of COVID-19 Information on the Relation between Frequency of COVID-19 Information on (a) Facebook, (b) TikTok, and (3) Twitter with Social Well-being**

(a)

(b)

(c)

*Note.* Solid lines indicate a significant effect and dashed lines indicate a non-significant effect.
